# Cross-Species Comparison of Metabolomics to Decipher the Metabolic Diversity in Ten Fruits

**DOI:** 10.3390/metabo11030164

**Published:** 2021-03-12

**Authors:** Jinwei Qi, Kang Li, Yunxia Shi, Yufei Li, Long Dong, Ling Liu, Mingyang Li, Hui Ren, Xianqing Liu, Chuanying Fang, Jie Luo

**Affiliations:** 1 School of Horticulture, Hainan University, Haikou 570288, China; qijinweihainan@163.com; 2 School of Tropical Crops, Hainan University, Haikou 570288, China; kang_li@hainanu.edu.cn (K.L.); yunxiashi225@163.com (Y.S.); ling.liu@hainanu.edu.cn (L.L.); lygaugau@163.com (M.L.); liuxq@hainanu.edu.cn (X.L.); 3 National Key Laboratory of Crop Genetic Improvement and National Center of Plant Gene Research (Wuhan), Huazhong Agricultural University, Wuhan 430070, China; yf.li@webmail.hzau.edu.cn; 4 Horticultural Research Institute, Guangxi Academy of Agricultural Sciences, Nanning 530007, China; donglong3127@163.com (L.D.); renhui0988@163.com (H.R.)

**Keywords:** fruit, nutrient, non-targeted metabolomic analyses, metabolome

## Abstract

Fruits provide humans with multiple kinds of nutrients and protect humans against worldwide nutritional deficiency. Therefore, it is essential to understand the nutrient composition of various fruits in depth. In this study, we performed LC-MS-based non-targeted metabolomic analyses with ten kinds of fruit, including passion fruit, mango, starfruit, mangosteen, guava, mandarin orange, grape, apple, blueberry, and strawberry. In total, we detected over 2500 compounds and identified more than 300 nutrients. Although the ten fruits shared 909 common-detected compounds, each species accumulated a variety of species-specific metabolites. Additionally, metabolic profiling analyses revealed a constant variation in each metabolite’s content across the ten fruits. Moreover, we constructed a neighbor-joining tree using metabolomic data, which resembles the single-copy protein-based phylogenetic tree. This indicates that metabolome data could reflect the genetic relationship between different species. In conclusion, our work enriches knowledge on the metabolomics of fruits, and provides metabolic evidence for the genetic relationships among these fruits.

## 1. Introduction

Nutritional deficiency threatens over 3 billion people [1] and leads to a series of health problems. A lack of vitamins results in many disorders [2]. Vitamin A deficiency increases the incidence rate and mortality of infectious diseases and causes night blindness, which threatens 125–130 million children living in developing countries [3]. The world-wide deficiency of folic acid (vitamin B9) may lead to pellagra, birth defects, and cardiovascular problems. Additionally, other vitamin deficiencies can also cause various human disorders [4]. Moreover, some secondary metabolites, such as flavonoids and polyphenols, are health-enhancing substances. They are bioactive in antioxidants, anti-atherosclerotic, anti-inflammatory, antitumor, anti-thrombogenic, anti-osteoporotic, and antiviral [5,6,7].

Food choices are closely related to human health [8]. With the popularization of knowledge about the dietary structure, the awareness of fruit’s benefits in alleviating chronic diseases is becoming more profound. The Dietary Guidelines encourage the consumption of three or more fruits and vegetables a day to prevent cardiovascular disease and heart disease [9,10]. Therefore, it is pivotal to clarify the kinds and amounts of nutrients in each fruit species.

The integration of diverse metabolomics approaches deepens our understanding of plant metabolic diversity. Matsuda et al. [11] performed a study with gas chromatography-time of-flight-mass spectrometry (GC-TOF-MS), capillary electrophoresis-time-of-flight-mass spectrometry (CE-TOF-MS), liquid chromatography-ion trap time-of-flight-mass spectrometry (LC-IT-TOF-MS), and liquid chromatography-quadrupole-time-of-flight-mass spectrometry (LC-Q-TOF-MS) techniques. They detected a total of 759 compounds in rice grains, including amino acids, sugars, fatty acids, and flavonoids. Many metabolomics studies have contributed to comprehending the intra-species diversity of metabolites. A multi-omic study has revealed that tomato cultivars accumulate less steroidal glycoalkaloids than ancient specimens. Meanwhile, the peel color varied across tomato accessions for a distinct amount of colorful metabolites [12]. Furthermore, efforts have been made to elucidate the metabolic signatures of fruits from distinct yet related species. For instance, pummelo and grapefruit accumulate less methoxylated flavonoids than their relatives, including lemon, mandarin, and orange [13]. The development of plant metabolomics contributes to understanding the fruit metabolome [14,15,16]. However, the metabolic diversity of fruits with less phylogenetic relatedness has not yet been sufficiently investigated. Lim et al. investigated metabolomic profiling with indigenous Australian fruits. Their work uncovered the nutritional potential of these Australian bush fruits [17]. This also suggests that it is essential to perform comparative studies on metabolomics with a broader range of fruits.

Plant metabolites are essential for plant growth, development, evolution, and adaptation to changing environments [18]. Metabolomics may provide a new strategy for understanding plant evolution and crop breeding. Recently, a comparative metabolomics study offered new insights into the differentiation in maize and rice [19]. However, the metabolic basis underlying the differential evolution of fruits remains elusive.

Many younger fruit species present in the market are famous for their variable bioactivity [20,21,22,23,24]. However, the metabolic differences between younger and older fruits still need to be explored. In this study, we carried out a comparative metabolic profiling analysis with fruit from ten species with less phylogenetic relatedness. The species included apple (*Malus domestica* Borkh.), grape (*Vitis vinifera* L.), mandarin orange (*Citrus reticulata* Blanco.), strawberry (*Fragaria × ananassa* D.), mango (*Mangifera indica* L.), starfruit (*Averrhoa carambola* L.), mangosteen (*Garcinia mangostana* L.), guava (*Psidium guajava* L.), blueberry (*Vaccinium corymbosum* L.), and passion fruit (*Passiflora edulis* Sims.). The first five species represent older fruits, while the other five are younger. Our work identified rich metabolic diversity across the ten fruits through a comparative analysis of metabolomics data and provided metabolic evidence for these fruits’ genetic relationship.

## 2. Results

### 2.1. Metabolomics Analysis of Ten Fruits

**Figure 1:**
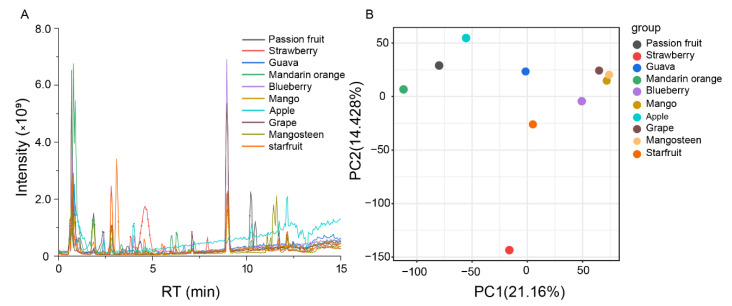
Analysis of metabolic variation in ten fruits using Q Exactive Focus Orbitrap LC-MS/MS. (**A**) Total ion chromatography of metabolites in ten kinds of fruits. (**B**) Principal component analysis (PCA) of the total ion chromatography of ten kinds of fruits.

To underpin the metabolic diversity across fruit species, we selected ten fruits for further study. These included passion fruit, strawberry, guava, orange, blueberry, mango, apple, grape, mangosteen, and starfruit. We carried out LC-MS-based non-targeted metabolomics and detected 20,775 metabolic signals (Figure 1A). Among them, 8443 were detected in passion fruit, 8168 in strawberry, 7088 in guava, 9304 in mandarin orange, 6358 in blueberry, 5701 in mango, 7829 in apple, 5642 in grape, 6403 in mangosteen, and 7981 in starfruit.

To better understand the metabolic variation between these species, we first compared the similarities and differences of metabolites in different species. Although the ten species shared 3199 metabolic signals, 703, 936, 489, 1043, 315, 245, 440, 100, 519, and 660 metabolic signals were specifically present in passion fruit, strawberry, guava, orange, blueberry, mango, apple, grape, mangosteen, and starfruit (Table S1).

Next, we performed a principal component analysis (PCA) of all samples based on the LC-MS data. PCA revealed that components 1 and 2 explained 21.16% and 14.42% of the variability, respectively (Figure 1B). Since grape, mangosteen, and mango clustered together in PCA score plots, these three fruits’ metabolic diversity is non-significant. Meanwhile, components 1 and 2 successfully separated the other fruits, indicating significant metabolic diversity.

### 2.2. Identification of Metabolic Signals

To gain insight into metabolites in different species, we characterized these metabolic signals. We produced each metabolic signal with the retention time (RT), relative abundance of fragments, and mass loss during fragmentation. Then, we screened the data in the literature and databases (such as MassBank [25] and METLIN [26]) using these features. Additionally, for several crucial metabolites, we annotated them with the help of standard information.

**Figure 2:**
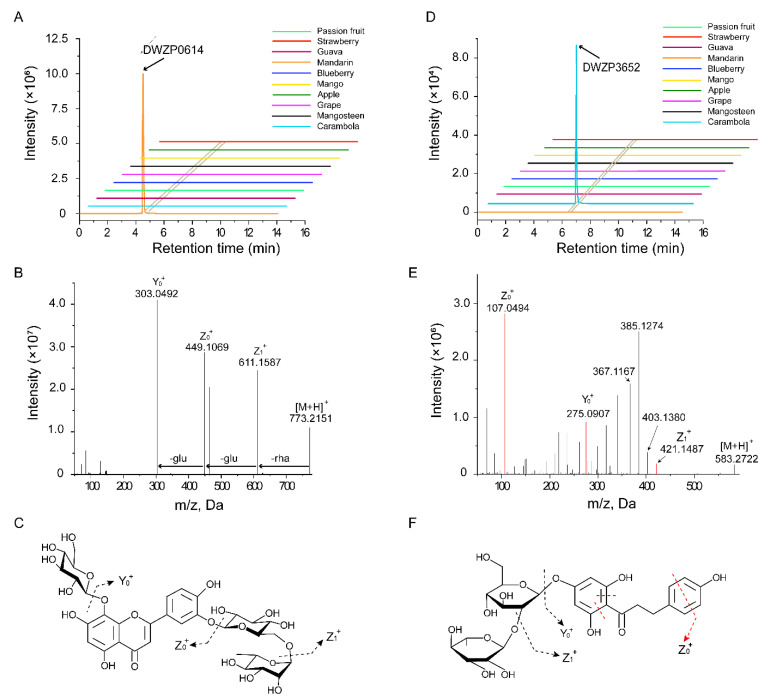
Detection and identification of specific metabolite signs by Q Exactive Focus Orbitrap LC-MS/MS. (**A**) Extracted ion chromatogram (EIC) of DWZP0614 at 4.50 min. DWZP0614 is unique in citrus. (**B**) MS/MS spectra of DWZP0614 at *m/z* 773.2151. The metabolite was identified as 8-hydroxyluteolin 8-glucoside-3’-rutinoside. (**C**) The molecular structure of the 8-hydroxyluteolin 8-glucoside-3’-rutinoside and its general fragmentation rules. (**D**) EIC of DWZP3652 at 6.24 min. DWZP3652 is unique in carambola. (**E**) MS/MS spectra of DWZP3652 at *m/z* 583.2722. The metabolite was identified as naringin dihydrochalcone. (**F**) The molecular structure of the naringin dihydrochalcone and its general fragmentation rules.

For example, DWZP0614 and DWZP3652 specifically existed in mandarin and starfruit, respectively (Figure 2A,D). DWZP0614 (RT 4.79 min) yielded a precursor ion [M + H]^+^ at *m/z* 773.2151. The tandem mass spectrum of DWZP0614 showed a high intensity fragment Y_0_^+^ ion at *m/z* 303.0492, which indicated the presence of a hydroxyluteolin-derived skeleton. The Z_1_^+^ ion at *m/z* 611.1587 [M + H − 162]^+^ and Z_0_^+^ ion at *m/z* 449.1069 [M + H − 162 − 162]^+^ both corresponded to the loss of a molecule hexose moiety. Moreover, a further loss of 162 Da produced Y_0_^+^ based on Z_0_^+^, which proved that there was a third hexose moiety in DWZP0614. Therefore, we characterized DWZP0614 as 8-hydroxyluteolin 8-glucoside-3’-rutinoside (Figure 2B,C).

DWZP3652 (RT 6.20 min) showed a precursor ion [M + H]^+^ at *m/z* 583.2722, and displayed a series of major fragment ions at *m/z* 275.0907 (Y_0_^+^) and abundant ions at 107.0494 (Z_0_^+^), which indicated that compound DWZP3652 was a derivative of phloretin. The elimination of 162 Da from the C position produced a fragment ion at *m/z* 421.1487 (Z1^+^) [M + H − 162]^+^, and a further loss of 146 Da from the O position continued to produce Y_0_^+^ [M + H − 162 − 146]^+^ on the basis of Z1^+^, which indicated that DWZP3652 was a dihexosidic derivative of phloretin. The fragment ions at *m/z* 403.1380, *m/z* 385.1274, and *m/z* 367.1167 resulted from the successive elimination of H_2_O (18 Da) from Z_1_^+^. Eventually, by comparing the data with those of the database, we named the sample naringin dihydrochalcone (Figure 2E,F).

In total, we annotated 2597 high quality (S/N > 10) metabolic signals in ten fruits, including 163 flavonoids, 49 amino acids and their derivatives, 39 chalcones, 14 lipids, and 8 vitamins (Table S2).

### 2.3. Metabolites’ Accumulation Pattern of Ten Different Fruits

**Figure 3:**
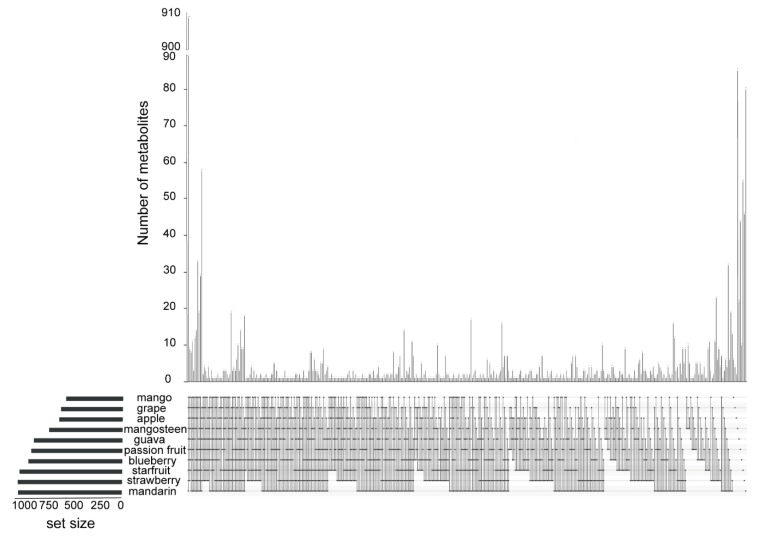
An upset plot of the number of metabolites detected in ten kinds of fruits. Black dot(s) at the bottom of each vertical bar indicates the intersection, which is made up of fruits that share the same metabolite. The lined dots indicate that two or more fruits shared the same metabolites. The black vertical bars at the top of the diagram indicate the number of metabolites of the corresponding intersection. The total numbers of metabolites detected in each fruit are represented by horizontal bars on the left.

We conducted a comparative analysis to underpin the accumulation pattern of metabolites across the ten species. Although the ten fruits shared 909 metabolites, passion fruit, mango, mangosteen, guava, starfruit, mandarin orange, apple, grape, blueberry, and strawberry had 44, 6, 86, 22, 55, 80, 2, 4, 10, and 46 species-specific metabolites, respectively (Figure 3).

To further identify metabolites’ content diversity, we quantified the metabolites by scheduled multiple reaction monitoring (sMRM). In total, 297, 106, 273, 170, 262, 499, 109, 46, 154, and 313 metabolites accumulated with the highest relative content in passion fruit, mango, mangosteen, guava, starfruit, mandarin orange, apple, grape, blueberry, and strawberry, respectively. Meanwhile, the relative content of 121, 414, 308, 88, 83, 99, 305, 329, 140, and 46 metabolites was the lowest in passion fruit, mango, mangosteen, guava, starfruit, mandarin orange, apple, grape, blueberry, and strawberry, respectively (Table S3).

### 2.4. Metabolic Profiling Analyses of Ten Different Fruits

**Figure 4:**
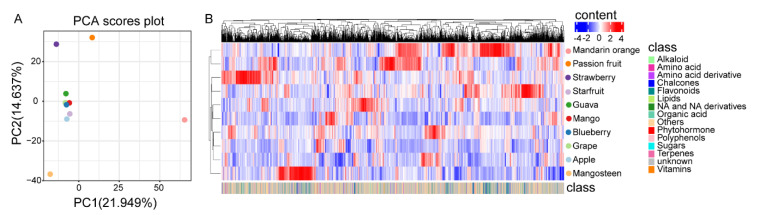
Metabolic variation of the ten kinds of fruits. (**A**) Principal component analysis (PCA) of the metabolite profiling of ten fruits. The mean value of three biological replications was used for PCA. (**B**) Heat map based on metabolome data of ten kinds of fruits. The mean value of three biological replications was used for metabolite profiling. The content value of each metabolite was normalized, and hierarchical clustering was performed. The red color indicates a high abundance of a metabolite, whereas the blue color represents a low relative abundance of a metabolite. Each fruit species is visualized in a single row, and each metabolite is represented by a single column. The bottom annotation with different colors represents the class to which the corresponding metabolite belongs.

To further investigate the metabolic variation across the ten species, we analyzed the metabolites’ accumulation patterns. PCA revealed that components 1 and 2 explained 21.9% and 14.6% of the variability, respectively (Figure 4A). As shown in the PCA score plots, passion fruit, mangosteen, and mandarin orange were entirely separated from the other fruits. Simultaneously, we found less separation among mango, guava, starfruit, apple, grape, and blueberry, which indicated the metabolic diversity among different fruits. The species-dependent accumulation pattern was further visualized by a heatmap based on the ten fruits’ metabolome data. As shown by hierarchical clustering based on the metabolomes, the metabolic diversity in different fruits was further supported (Figure 4B).

### 2.5. Comparative Analysis of Accumulation Patterns of Secondary Metabolites in Ten Fruits

**Figure 5:**
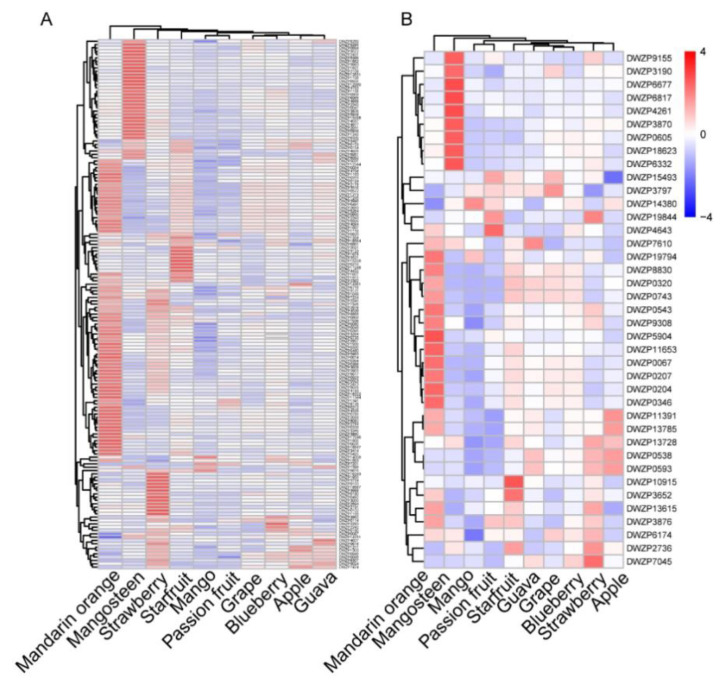
The accumulation pattern of different flavonoids and chalcones in different kinds of fruits. (**A**) Heat map of the metabolic diversity of flavonoids in ten kinds of fruit. (**B**) Heat map of the metabolic diversity of chalcones in ten kinds of fruit. The mean value of three biological replications was used for metabolite profiling. The content value of each metabolite was normalized, and hierarchical clustering was performed. Each fruit species is visualized in a single column, and each metabolite is represented by a single row.

The accumulation of secondary metabolites, such as flavonoids and chalcones, was significantly different among the ten fruits. Visualization of the flavonoid profile by hierarchical cluster analysis (HCA) displayed apparent phenotypic variation in their relative abundance in four fruits. Compared with the other six fruits, mandarin orange, mangosteen, strawberry, and starfruit had a higher relative content of flavonoids—72, 36, 22, and 12 flavonoids with the highest relative contents, respectively. However, mango accumulated the lowest content of most flavonoids (Figure 5A and Table S3). To further elucidate the metabolic diversity of flavonoids in the ten fruits, we analyzed the accumulation pattern of chalcones, which are precursors of flavonoid synthesis, in the ten fruits. As shown by HCA based on the chalcones, mandarin orange, mangosteen, strawberry, and starfruit also have higher relative levels of chalcone compared to the other fruits, with 14, 9, 5, and 2 chalcones with the highest relative contents, respectively (Figure 5B and Table S3).

### 2.6. The Metabolome Has the Potential to Reflect the Evolutionary Relationships between Fruits

**Figure 6:**
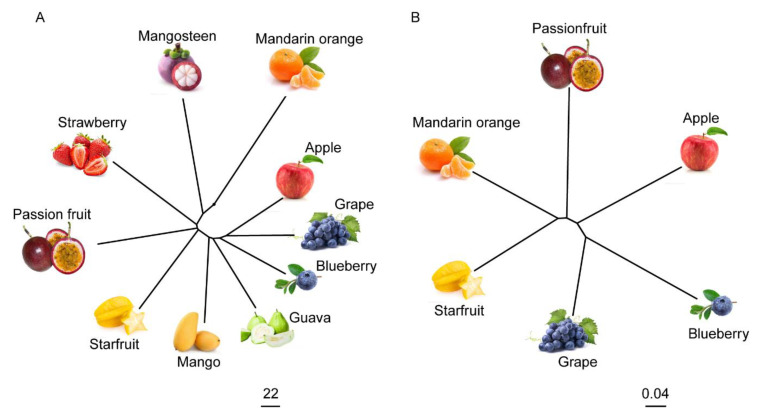
Phylogenomic relationships of different kinds of fruits. (**A**) The neighbor-joining tree of the ten fruit species with metabolome data. The scale bar indicates the simple matching distance. (**B**) The phylogenomic analysis of six fruit species. A phylogenetic tree was constructed using the single-copy protein data of passion fruit, apple, blueberry, grape, starfruit, and mandarin orange. The scale bar indicates the simple matching distance.

A neighbor-joining tree using metabolome data of the ten fruits was constructed (Figure 6A). Meanwhile, we also created a phylogenetic tree using the single-copy protein data of passion fruit, apple, blueberry, grape, starfruit, and mandarin orange (Figure 6B). Apple, grape, and blueberry were closely clustered in the metabolome-based and single-copy protein-based trees. As shown in the phylogenetic trees, passion fruit, starfruit, and mandarin orange were progressively more distantly related to apple, grape, and blueberry (Figure 6). These results indicate that the metabolomes of different fruits could reflect a close genetic relationship between different fruits to a certain extent.

## 3. Discussion

Fruits play an essential role in the human diet because of their health-promoting properties [27,28]. However, the energy and nutrients vary significantly in different fruits. Therefore, the metabolome of different fruits could help humans to maintain a well-balanced diet to meet the nutrient needs and provide new insights for fruit breeding. In recent years, the rapid development of analytical approaches has accelerated plant metabolic studies [29,30,31,32,33]. The LC-MS-based non-targeted profiling approach has been an effective method in the investigation of plant metabolism. For instance, Wang et al. detected metabolites of different citrus tissues by non-targeted LC-MS. They found differential accumulation patterns of both flavonoids and amino acids in various tissues and species [13]. Although metabolomics-based methods have been applied in fruits [13,29,34,35,36], comparative analyses of nutritional metabolites in distinct fruits are rarely conducted. In this study, we detected more than 20,000 metabolic signals with the non-targeted LC-MS method. We identified more than 300 metabolites, including primary metabolites, such as lipids, vitamins, amino acids, and secondary metabolites (Table S1).

The accumulation of metabolites varied in different species and varieties [37,38,39,40,41,42]. The metabolome of the ten fruits showed significant differences. Although about half of the metabolites were detected in all fruits, some were species-specific; that is, humans need diversified fruits to meet the nutrient needs.

Flavonoids are essential for human health, and are bioactive in anti-atherosclerosis, anti-inflammatory, anti-allergy, antibacterial, anti-tumor, and anti-oxidative [5,43,44,45,46,47,48,49,50]. Fruits are rich in flavonoids, and different kinds of flavonoids have been identified in fruits [13,51,52]. The accumulation of flavonoids displays tissue specificity and natural variation in fruits [7,13,53,54]. Therefore, underpinning the diversity of flavonoids in different fruits is critical for human health. In this study, we found that mandarin orange showed the highest relative flavonoid content, followed by mangosteen and strawberry, which contained 72, 36, and 22 kinds of flavonoids with the highest relative levels, respectively. The relative content of flavonoids was relatively low in other fruits, especially in mango.

Metabolites, which are the end products of various biological processes, are the material bases of gene phenotypes and have the potential to act as accurate biomarkers for upstream biological events [55,56]. Therefore, closely related species would present similar metabolomes. In this study, we constructed phylogenetic trees using metabolome and genome data of different fruits. The genome-based phylogenetic tree showed close relationships among apple, blueberry, and grape, consistent with the metabolome-based evolutionary tree. Additionally, passion fruit, starfruit, and orange shared a distant relationship with these three fruits in single-copy protein-based phylogenetic trees. Orange had the weakest relationship with these three fruits, which was consistent with the results of metabolome-based phylogenetic trees (Figure 6). This result confirmed that metabolomes can reflect the evolutionary relationship between plants in the absence of genomes.

## 4. Materials and Methods

### 4.1. Plant Materials

To study the differences in metabolites among multiple species, we selected ten kinds of fruits that are very common and popular. The ten fruits included passion fruit, mango, starfruit, mangosteen, guava, strawberry, mandarin orange, blueberry, apple, and grape. In the growing seasons of 2019–2020, passion fruits (*P. edulis*, a cultivar of purple passion fruit), mangos (Alphonso, a traditional Indian cultivar), starfruits (Malaysia B17, a popular diploid carambola cultivar), mangosteens (dark purple, a cultivar introduced from Thailand), and guavas (New Age, a cultivar whose genome has been reported) from six healthy trees in the breeding base of Hainan University were randomly sampled; blueberries (Northland) were harvested in July 2018 from the Shenyang Crown Blueberry Biotechnology; strawberries (Camarosa, one of the varieties with the largest planting area in the world) and mandarin orange (Tribute Citru, a popular cultivar in China) from six healthy plants in the College of Horticulture and Forestry, Huazhong Agricultural University, Wuhan, were randomly sampled; and apples (Royal Gala) and grapes (Pinor Vermei, one of the most popular grape varieties in the world) from six healthy trees in the breeding base of Shandong Agricultural University were randomly sampled. Samples were harvested and frozen in liquid nitrogen. Three biological replicates were collected from each fruit.

### 4.2. Chemical Reagents

Chromatographic-grade acetonitrile, acetic acid, and methanol were purchased from Merck (Darmstadt, Germany). The water used as Milli-Q water was purified using a Millipore purification system (Millipore Corporation, Burlington， MA, USA). In this study, the standard lidocaine was bought from Shanghai New Asiatic Pharmaceuticals Co., Ltd. (Tianjin, China). All standards used in tests were stored in a −20 °C refrigerator in darkness.

### 4.3. Metabolite Sample Preparation

Three biological replications of these ten fruits were collected, solidified with liquid nitrogen, and stored at −80 °C until the metabolomics analysis. The samples were lyophilized and ground into powder using a mix mill (MM400, Retsch) with a zirconia bead for 1 min at 30 Hz. Then, 100 mg powder was weighed and 70% methanol aqueous solution was added to 0.1 mg mL^−1^. Next, ultrasonication was used to extract the sample mixture at 40 Hz for 10 min, which was centrifuged and filtered [57,58]. Then, the MRM method of LC-MS 8060 was used to quantify the metabolites of the mixture, and the detection window was set to 120 s and a target scanning time of 1.5 s. The original data obtained by the instrument were processed by Insight software. In order to improve the normalization, the relative signal strength of the metabolite was divided and normalized according to the internal standard (0.1 mg L^−1^ lidocaine), and log 2 was then used to transform the value.

### 4.4. Metabolite Profiling

Each sample was performed in the Full-Scan mode by Q Exactive Focus Orbitrap LC-MS/MS (Thermo Scientific, Waltham, MA, USA) using Compound Discoverer 3.1 software to analyze the raw data. The quantification of metabolites was carried out in the multiple reaction monitoring (MRM) mode using LC-MS 8060 (Shimadzu, Japan). The analytical conditions were as described previously [58]. Qualitative and quantitative chromatographic conditions were consistent.

The detection of material metabolites, retention time, mass-to-charge ratio, and MS/MS2 of all detectable ions were recorded. The ion characteristics of the sample were automatically matched with the internally established reference libraries of chemical standard entries to identify metabolites. Metabolic differences between these four different fruits were determined using nested ANOVA in the R package. In addition, the metabolite profiles were subject to principal component (PC), and network-based analyses, with the latter being based on metabolite–metabolite and metabolite–morphological trait correlations employing the mean profile values.

### 4.5. Identification of Metabolites

For high-quality (S/N > 10) metabolic signals, we first compared the MS2 spectral information of metabolic signals with the database by using Compound Discover 3.1 software, and annotated these metabolic signals in batches. Then, we identified metabolic signals that did not match the information in the database by querying the MS2 spectral data taken from the literature or searched the databases (e.g., METLIN [26] and MassBank [25]). Additionally, for some compounds whose standards were available, this identification was carried out by a comparison of the accurate *m/z* values, the retention time (RT), and the fragmentation patterns with those obtained by injecting standards using the same conditions.

### 4.6. Phylogenomic Analysis of the Ten Fruits

The hierarchical clustering tree using metabolome data of the ten fruit species was constructed using the hclust packages in R (www.r-project.org/ (version 4.0.3) (accessed on 18 January 2021)). The ggtree of R software (www.r-project.org/ (accessed on 18 January 2021)) was used for visualizing the hierarchical clustering tree. The phylogenetic tree using the single-copy protein data of passion fruit, apple, blueberry, grape, starfruit, and mandarin orange was constructed using the RAxML software (http://phylobench.vital-it.ch/raxml-bb/ (version 8.2.12) (accessed on 18 January 2021)). The ggtree of R software (www.r-project.org/ (accessed on 18 January 2021)) was used for visualizing the phylogenetic tree. The protein sequences of apple, grape, and mandarin orange were extracted from the Phytozome database (http://phytozome.jgi.doe.gov/pz/portal.html (v13) (accessed on 18 January 2021)). The protein sequences of passion fruit, starfruit, and blueberry were downloaded from supporting information of literature [35,59,60]. 

## 5. Conclusions

In conclusion, we identified the metabolic diversity of ten fruits with less phylogenetic relatedness, supporting the necessity of diversified fruits. Our work enriches knowledge on the metabolomics of fruits, and provides metabolic evidence for the genetic relationship among these fruits.

## Data Availability

The blueberry raw sequence data were obtained from NCBI BioProject ID PRJNA494180 [61], and the complete genome was downloaded from the CoGe platform (https://genomevolution.org/coge/GenomeInfo.pl?gid=36464 (accessed on 18 January 2021)) [60]. The star fruit whole-genome sequence data were deposited in the https://bigd.big.ac.cn/gwh (accessed on 18 January 2021), under accession number GWHABKE00000000 [59]. The apple, mandarin orange, and grape genome reads were deposited under BioProject ID PRJNA379390 [62], PRJNA225968 [63], and PRJNA550461 [64], respectively. The genome data of passion fruit was described in previous work [35]. The data presented in this study are available in the supplementary material.

## Supplementary Materials

The following are available online at https://www.mdpi.com/2218-1989/11/3/164/s1: Table S1: Metabolic signals detected in the ten fruits, Table S2: Metabolites detected in the ten fruits, Table S3: Metabolite variation in the ten fruits.
